# Sucrose synthase dynamics and its potential role in heat stress tolerance in cereals

**DOI:** 10.3389/fpls.2025.1652076

**Published:** 2025-09-16

**Authors:** Priyanka Parihar, Jai Prakash Jaiswal, Ashok Kumar Verma, Amit Kumar

**Affiliations:** ^1^ Department of Genetics and Plant Breeding, Govind Ballabh Pant University of Agriculture and Technology, Pantnagar, Udham Singh Nagar, Uttarakhand, India; ^2^ Department of Biochemistry, Govind Ballabh Pant University of Agriculture and Technology, Pantnagar, Udham Singh Nagar, Uttarakhand, India

**Keywords:** carbohydrate metabolism, abiotic stress, starch, sink-source, sink strength

## Abstract

Sucrose synthase (SuSy) is a key enzyme in plant carbohydrate metabolism, catalyzing the reversible conversion of sucrose into UDP-glucose and fructose. SuSy is central to several developmental and metabolic processes, where its activity is closely linked to biomass accumulation, pollen viability, grain filling, and seed development. This review explores the role of SuSy, in comparison with invertase, examines its enzymatic interactions, and highlights its contribution to metabolic adaptation under heat stress, while emphasizing its critical role in strengthening sink capacity. Elevated temperatures negatively impact sucrose metabolism and source–sink relationships, disrupting yield formation in cereal crops. SuSy, with its distinct isoforms and subcellular localizations, adapts flexibly to thermal stress, maintaining sucrose flux and stabilizing energy supply in developing tissues. Its stress-responsive expression patterns suggest that specific isoforms could be targeted to enhance thermotolerance. Overall, understanding the spatial, temporal, and regulatory dynamics of SuSy offers promising avenues for developing climate-resilient crops. Harnessing its full potential through targeted breeding and gene editing could be pivotal in mitigating the adverse effects of rising temperatures on global food security.

## 1 Introduction

Sucrose is the principal form of transported carbohydrate in higher plants and plays a vital role in regulating growth, development, and stress responses. Its metabolism is tightly controlled to balance energy supply with the demands of various physiological processes. In plants, the cleavage of sucrose constitutes the major route of carbon flux, making it the primary pathway for carbon turnover (Ruan, 2014). This process is catalyzed either by invertases (INV) or by sucrose synthase (SuSy, EC 2.4.1.13). While INV catalyzes irreversibly, the SuSy enzyme has the unique capability to cleave as well as synthesize sucrose in a nearly energy-neutral way (Ruan, 2014; Kleczkowski and Decker, 2015). The relative activities of these enzymes determine the direction and efficiency of carbon partitioning and are critical for plant adaptation under various environmental conditions. Plants create a spatial and temporal system of sucrose sources and sinks, enabling effective sucrose transport, by controlling the activity levels of the enzymes involved in synthesis or cleavage at different stages of growth, across various plant organs, and within distinct cellular compartments. SuSy catalyzes a process that combines respiration, the production of carbohydrates, and the utilization of carbohydrates. This interaction makes it possible to quickly transform a sucrose sink, like the growing endosperm of cereals, into a sucrose source without requiring the production or breakdown of enzymes. Although INV plays an important role in normal plant growth, SuSy is particularly involved in processes such as pollen tube growth, the establishment of nitrogen fixation, biomass production, and the maturation of fruits and seeds, especially under abiotic stress conditions. SuSy is typically found in the cytoplasm of both photosynthetic and non-photosynthetic cells, including the vascular tissues of various plants, which suggests a potential role in sucrose translocation (Tomlinson et al., 1991; Geigenberger and Stitt, 1993; Chen et al., 2017). It was previously believed to be exclusively cytosolic; however, recent studies have revealed its presence in the cytoskeleton and tonoplasts, as well as in various organelles such as plastids, vacuoles, Golgi apparatus, and mitochondria. This diverse localization supports SuSy’s various functions, such as providing carbon for starch synthesis in plastids, facilitating solute exchange with mitochondria, and interacting with the cytoskeleton (Winter and Huber, 2000; Subbaiah et al., 2006). SuSy is a major carbohydrate-metabolizing enzyme, alongside ADP-glucose pyrophosphorylase (AGPase), sucrose phosphate synthase (SPS), sucrose phosphate phosphatase, soluble starch synthase (SSS), and starch branching enzyme (SBE), all of which are crucial for regulating the metabolic status of source leaves (Ruan, 2014). From source leaves, sucrose is transported to maintenance and storage sites based on the sink strength. Sink strength is one of the several characteristics that determine yield, especially in cereal crops. Sink organs have the capacity to import carbohydrates; for example, during wheat grain formation, the carbohydrate is transferred as sucrose and uploaded into growing grains, where it is converted into starch by enzymes. Therefore, the importance of enzymes related to sucrose metabolism and starch synthesis becomes all the more important, especially in the context of abiotic stresses. Plant response to a particular abiotic stress is a complex regulatory procedure involving roles played by enzymes, other biomolecules, and hormones and the crosstalk among them that facilitates providing a survival toolkit to the plant. This review explores the multifaceted role of SuSy, its comparison with INV, how SuSy activity changes during heat stress, and whether or not it is important to be considered for conducting studies revolving around heat stress faced by plants in general and cereal crops in particular.

## 2 Structure, function and evolution of sucrose synthase in higher plants

Sucrose synthase is an extensively characterized enzyme. It is encoded by a minor multigene family in the higher plants. SuSy genes of various species have been studied, and a conclusion regarding the structural conservation among them is drawn, while they still express differentially and depict functional divergence, alongside the evolution of this gene family. SuSy is a member of the broader metal-independent GT-B glycosyltransferase superfamily, specifically the retaining GT-4 subfamily. In both bacteria and plants, this enzyme usually exists as homotetramers (in some species it exists as heterotetramers), with each monomeric subunit having a molecular mass of approximately 90 kDa (Porchia et al., 1999; Wu et al., 2015). It may range between 53 and 110 kDa in different plant species. The proposed structure was established by the X-ray crystallography of SuSy of *Arabidopsis thaliana* (*AtSus1*) and *Nitrosomonas europaea* (Zheng et al., 2011; Wu et al., 2015), both of which have demonstrated structural conservation and a 50.3% identical sequence. A conventional SuSy encompasses two domains that are highly conserved: an N-terminal domain dedicated to sucrose synthesis, comprising roughly 550 amino acids, which facilitates cellular localization, and a C-terminal domain, consisting of approximately 175 amino acids, that exhibits glycosyltransferase activity (Zheng et al., 2011). These domains undergo phosphorylation at critical sites, which plays a significant role in the precise modulation of their functional activity. For further detailed explanation on the structure of SuSy, refer to Schmölzer et al. (2016). The phosphorylation at Ser 13 and Ser 167 modulates the biochemical properties of plant SuSy. Initial phosphorylation at Ser15 activates SuSy and primes it for further phosphorylation at Ser170, leading to ubiquitin-mediated degradation (Huber et al., 1996). The first phosphorylation, driven by Ca²^+^-dependent protein kinases (CDPKs) or a Ca²^+^-independent SnRK, is responsive to sugar levels (Zhang et al., 1999; Chikano et al., 2001). However, only CDPKs perform the second phosphorylation. ENOD40 proteins can prevent SuSy breakdown by blocking the second phosphorylation site, impacting vascular function, phloem transport, and assimilate flow, particularly in high-sink areas with active phloem unloading (Kouchi et al., 1999; Hardin et al., 2003).

**Figure 1:**
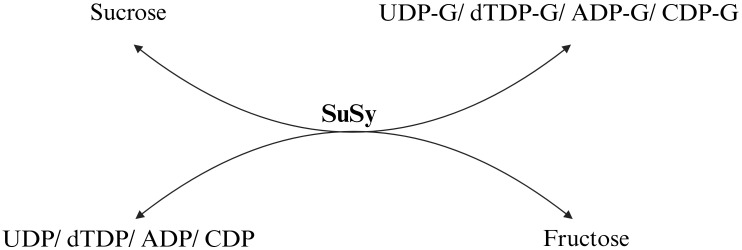
The reversible reaction catalyzed by sucrose synthase (SuSy). The enzyme shows the highest activity with UDP-glucose (UDP-G), followed by dTDP-glucose, ADP-glucose, and CDP-glucose, with relative activities of 100%, 26%, 17%, and 2%, respectively (Römer et al., 2004; Zheng et al., 2011).

SuSy catalyzes the reversible conversion of sucrose and nucleoside diphosphates (NDPs) into fructose and NDP-glucose, where N represents thymidine, uridine, guanosine, adenosine, cytidine, or inosine (**Figure 1**). Most studies have concluded UDP is the preferred substrate due to its role in producing UDP-glucose (UDPG) (Curatti et al., 2008), but ADP also functions effectively and produces ADP-glucose (Schmölzer et al., 2016; Zhang et al., 2019). A key feature of SuSy is its ability to catalyze the formation of glycosidic bonds in sucrose with an energy content of -29.3 kJ/mol (Neufeld and Hassid, 1963), making the reaction nearly as efficient as those involving nucleotide-activated sugars. This allows the reaction to be reversible, which makes it valuable for industrial applications, such as recycling UDPG in Leloir GT reactions (Ardevol and Rovira, 2015). However, in *in vivo* conditions, the reaction favoring sucrose breakdown is dominant over synthesis owing to decreased overall energy requirements (Geigenberger and Stitt, 1993; Lee and Jeon, 2020). The reaction equilibrium is pH-dependent, showing optimal activity for sucrose synthesis between pH 7.5 and 9.5, while lower pH values (5.5 to 7.5) favor the reverse reaction (Bieniawska et al., 2007; Almagro et al., 2012). Although plant SuSy enzymes have temperature optima between 40°C and 55°C, their stability decreases above 30°C. In contrast, bacterial SuSy exhibits higher temperature optima, ranging from 60°C to 80°C (Figueroa et al., 2013; Diricks et al., 2015). These temperature and pH sensitivities influence SuSy’s role in metabolic processes. SuSy is tightly regulated at both transcriptional and post-transcriptional levels. This regulation ensures that its expression and activity are modulated in response to various developmental cues and environmental conditions (Hu et al., 2024; Shah et al., 2025).

### 2.1 Sucrose synthase isoforms and their evolution

The SuSy gene is hypothesized to have emerged in proteobacteria or a shared progenitor of both proteobacteria and cyanobacteria, with the possibility that plants acquired it through cyanobacteria (Lunn, 2002). SuSy was discovered in 1955 in wheat germ (Cardini et al., 1955), but it was maize’s *Shrunken* (*Sh*) gene that was first cloned and sequenced (Werr et al., 1985). It is one of the three maize SuSy isoforms: SUS1, SH1, and SUS2 (previously called SUS3), which are encoded by the *Sus1, Sh1*, and *Sus2* loci, respectively (Carlson et al., 2002). *Sh1* and *Sus1* are paralogous genes containing 16 exons/14 introns and 15 exons/14 introns, respectively, having virtually identical structure with only a difference in the last intron (Shaw et al., 1994). *Sus2*-*Sus1* forms hetero-oligomers, and *Sh1* exists as a homo-oligomer in maize kernel (Duncan et al., 2006). Orthologous genes corresponding to *Sh1* and *Sus1* have been reported in both barley and hexaploid wheat (Martinez de Ilarduya et al., 1993; Volpicella et al., 2016). In barley, these genes are located on chromosomes 7HS and 2HS, respectively (de la Hoz et al., 1992). In wheat, the *Sus1* and *Sus2* genes have been mapped to the short arms of chromosomes in homoeologous group 7 (Maraña et al., 1988). Additionally, a partial sequence of the *Sus3* gene has been identified in wheat (Mukherjee et al., 2015). Notably, the expression profiles of these genes differ between tissues: *Sus2* is specifically expressed in the endosperm, whereas *Sus1* transcripts are detected in roots and leaves (Maraña et al., 1990; Martinez de Ilarduya et al., 1993). The number of SuSy genes can differ among plant species, with two genes found in *Amborella trichopoda* (Zhang et al., 2013); five in grapes (Zhu et al., 2017), pomegranate (Liu and Zheng, 2022), litchi (Wang et al., 2021), sorghum (Lu et al., 2022), and sugarcane (Noman et al., 2022); and six in Arabidopsis (Baud et al., 2004), rice (Hirose et al., 2008), tomato (Goren et al., 2017; Duan et al., 2021), rubber tree (Xiao et al., 2014), cacao (Li et al., 2015), peach (Zhang et al., 2015), citrus (Islam et al., 2014), *Nicotiana sylvestris* (Wang et al., 2015), pineapple (Wu et al., 2024), and kiwi fruit (Liao et al., 2022). Meanwhile, seven SuSy genes are found in cotton (Chen et al., 2012), bamboo (Huang et al., 2018), and *Nicotiana tomentosiformis* (Wang et al., 2015). In apple, eleven SuSy genes have been identified (Tong et al., 2018); twelve in *Glycine max* (Xu et al., 2019); fourteen have been discovered in *Nicotiana tabacum* (Wang et al., 2015) and Brassica juncea (Koramutla et al., 2019; Li et al., 2021); and fifteen in poplar (Zhang et al., 2011) and *Dendrobium catenatum* (Jiang et al., 2022). Cultivated sweet potato contains nine SuSy genes, with seven each in its wild diploid relatives *I. trifida* and *I. triloba* (Jiang et al., 2023; Hu et al., 2024). Chinese pear has at least thirty SuSy genes (Abdullah et al., 2018), though at least five of these genes are likely non-functional due to incomplete SuSy and GT domains. Due to the presence of multiple isoforms, Susy genes exhibit varied tissue-specific functions and differential expression patterns depending on the developmental stage. For example, in peas, *SuSy1* is expressed in seeds, *SuSy2* in leaves, and *SuSy3* in floral tissues. Mutational studies, such as those involving the *rug4* (rugosus) mutant, demonstrate that the absence of *SuSy1* leads to phenotypic consequences that are not alleviated by the presence of *SuSy2* or *SuSy3*, underscoring the non-redundant roles of these isoforms. However, partial redundancy (as noted by studies such as Bieniawska et al., 2007; Zheng et al., 2011) and slight functional compensation by the VIN gene (Wan et al., 2018) can make it difficult to find individual genes. Now whether such functional divergence occurred independently across lineages or has its origins in early angiosperm evolution remains an open question (Thirugnanasambandam et al., 2019; Zhu et al., 2017).

The evolutionary information about this gene family remains largely unexplored, as most of these were derived from studies on individual angiosperm species. SuSy genes in plants form a monophyletic group, indicating their origin from a common ancestor. Phylogenetic analyses based on evolutionary relationships and distinct intron–exon architectures have categorized them into three anciently diverged subfamilies: SUS I, SUS II, and SUS III. Evidence suggests that SuSy genes evolved independently in monocots and dicots, with whole genome duplication events significantly influencing their diversification. The three subfamilies display varied expression profiles across plant species, implying that functional divergence likely preceded the monocot-dicot split. Among these, the SUS I and SUS II genes are more evolutionarily conserved, exhibiting broader expression across tissues and maintaining similar intron–exon structures. In contrast, SUS III genes appear to have undergone relaxed purifying selection, enabling them to develop novel functions and show more tissue-specific expression. Notably, even within monocots like rice, SUS III genes retain such tissue-specific patterns. The GT-B domain, associated with catalytic activity, is more conserved across SuSy genes compared to the regulatory domains, suggesting that these regions have been subject to different selective pressures (Xu et al., 2019). Further phylogenetic reconstruction, including gymnosperms, by Stein and Granot (2019), revealed early duplication events, highlighting the evolutionary trajectory of SuSy genes before the divergence of angiosperms and gymnosperms. SuSy shares similarities with SPS and glycogen synthases, with multiple isoforms found across different tissues. These isoforms have 50-70% similarity with each other but less than 25% with SPS. The study of SuSy gene families in plants has been greatly enhanced by advances in genome sequencing, assembly, and annotation. While molecular genetic studies have significantly advanced our understanding of the functions of individual proteins, evolutionary analyses offer the potential to provide deeper insights into the origins and diversification of the SuSy gene family, revealing further functional implications (Huang et al., 2021).

## 3 Role of sucrose synthase in plant growth

Sucrose synthase is a key player in sugar metabolism and regulates sucrose flux. Sucrose metabolism is crucial for development, yield production, and stress adaptation, primarily by producing various sugars that serve as energy sources and building blocks for the synthesis of vital compounds (Xiao et al., 2024; Aluko et al., 2021). Sucrose, primarily synthesized in mature leaves, can also be resynthesized within sink tissues. Its synthesis and degradation play important roles in maintaining energy balance, as enzymatic cleavage of sucrose into hexoses provides essential carbon and energy for the growth and development of sink organs. Physiological conditions and an increase in sucrose concentration in the storage and vascular tissues favor SuSy to cleave sucrose rather than synthesize it (Verma et al., 2019). SuSy is soluble in the cytoplasm and contributes readily to an adenylate-conserving path of respiration and for starch synthesis (Xu et al., 1989; Thirugnanasambandam et al., 2019). However, it can also associate and dissociate quickly from membrane and cytoskeletal sites, suggesting additional roles at the plasma membrane and Golgi apparatus related to cell wall formation (Hong et al., 2001; Lampugnani et al., 2018). It also has a possible role at the tonoplast related to the use and/or storage of vacuolar sucrose (Fugate et al., 2019). SuSy has been identified as an actin-binding protein, and this association presumably promotes plastid proximity, thereby facilitating starch biosynthesis (Winter and Huber, 2000; Kunjumon et al., 2024). SuSy also transiently associates with the plasma membrane-bound cellulose synthase complex, enabling the direct channeling of UDPG into cellulose biosynthesis, with concurrent recycling of UDP (Schneider et al., 2016). Cellulose production is crucial for forming secondary cell walls in xylem tissues, which contribute to mechanical support and overall structural integrity in plants. Furthermore, the subcellular localization of SuSy appears responsive to sugar signals and other metabolic cues, suggesting a regulatory mechanism that fine-tunes the enzyme’s role between biosynthetic and respiratory functions (O’Leary and Plaxton, 2018). Although SuSy exhibits general membrane affinity, evidence indicates that its membrane association is influenced by reversible phosphorylation at a conserved serine residue (Takeda et al., 2017; Mareri et al., 2021). This regulation is supported by kinase activity that responds to cellular signaling pathways (Chikano et al., 2001).

In maize, *Sh1* provides UDPG for the cell wall synthesis during the developmental phase of kernels; however, *Sus2* is highly expressed in various tissues and differs from *Sus1* and *Sh1* by lacking membrane association, suggesting a unique role in cytoplasmic sucrose degradation (McCarty et al., 1986; Chourey et al., 1998). In rice, *OsSUS1* shows high expression levels in the internodes, and its expression pattern closely aligns with that of cellulose synthase genes (Hirose et al., 2014). Elevated expression of *OsSUS3* has also been associated with increased accumulation of structural carbohydrates, including cellulose and hemicellulose (Cho et al., 2011; Fan et al., 2019). SuSy also plays a role in the formation of mixed-linkage poly-glycans and in the production of callose near the phragmoplast or in localized exoplasmic zones (Stass and Horst, 2009; Nedukha, 2015). In Arabidopsis, *AtSUS5* and *AtSUS6* exhibit phloem-specific expression and are functionally involved in the synthesis of callose (Bieniawska et al., 2007). Callose synthesis is instrumental in the assembly of sieve plates and plasmodesmata, both of which are vital for nutrient transport. Research indicates that genes in the SUS III clade may also be involved in the vascular development for differentiating xylem in higher plants, possibly coevolving with tissue-specific expression patterns. In young maize roots, both *Sh1* and *Sus1* are mainly expressed within the vascular cylinder (Chen and Chourey, 1989). In transgenic tobacco plants carrying the GUS reporter gene under the control of the maize *Sh1* promoter, *Sh-GUS* activity was specifically observed in phloem cells, with no detectable expression in other vegetative tissue cell types (Yang and Russell, 1990). Also, QTL analyses in maize endosperms have now provided the genetic evidence highlighting SuSy’s role in starch production and determining sink strength in heterotrophic organs (Thévenot et al., 2005). This significant role in phloem unloading and modulation of sink strength ensures that non-photosynthetic tissues receive sufficient sucrose necessary for their metabolic requirements and helps stabilize membranes and proteins under abiotic stresses (Julius et al., 2017; Durand et al., 2018). A knockout of *AtSUS2* and *AtSUS3* in Arabidopsis led to a reduction in starch accumulation during the early- to mid-developmental stages (Angeles-Núñez and Tiessen, 2010). In rice, both *OsSUS1* and *OsSUS3* are involved in seed starch accumulation and cell division to enhance grain weight and husk size. There was significant starch reduction in *sh1* maize mutants (Chourey and Nelson, 1976; Duncan et al., 2006), a 26% decrease in carrot (Tang et al., 1999), and a 34–63% reduction in genetically modified potato tubers (Zrenner et al., 1995) due to reduced SuSy activity.

**Figure 2:**
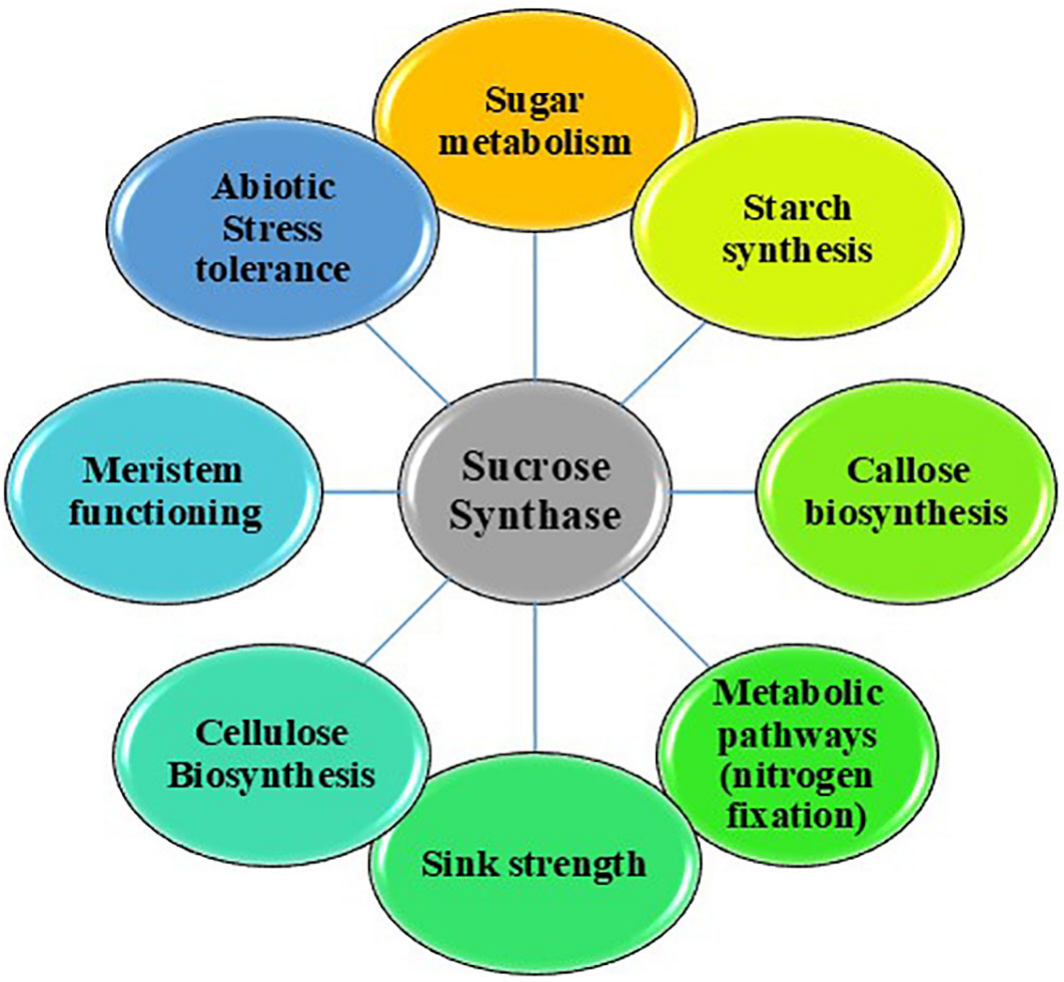
Multifaceted role of sucrose synthase.

In Arabidopsis, SuSy is localized within the companion cells located in the silique wall during the final phases of seed maturation, indicating that its activity in the embryo could utilize sucrose to generate precursors necessary for the synthesis of storage proteins and lipids (Fallahi et al., 2008). Similar patterns of localization have been observed in citrus fruits (Nolte and Koch, 1993) and radish hypocotyls (Rouhier and Usuda, 2001), indicating that SuSy may also contribute to energy supply for phloem loading and unloading in these tissues (Yao et al., 2020). Additionally, SuSy is associated with various developmental processes, including the functioning of meristems, where it may affect sugar and hormonal signaling pathways that are indispensable for growth and development. This gene is among the earliest to exhibit increased expression during the differentiation of leaf primordia from the apical meristem and has a role in auxin signaling (Pien et al., 2001; Chaudhary and Singh, 2024). In cucumber, the down-regulation of *Susy4* impedes the growth and development of fruits and flowers (Fan et al., 2019). A 70% suppression of SuSy activity in the ovule epidermis led to a fiberless phenotype in cotton (Ruan et al., 2003). Potato tubers with elevated levels of SuSy show notable agronomic benefits, including a marked increase in antioxidant activity and enhanced resistance to enzymatic browning compared to non-modified tubers. This reduction in browning is believed to result from SuSy’s role in safeguarding UDPG, which is crucial for the glycosylation and stabilization of polyphenolic compounds (Bahaji et al., 2014). Furthermore, SuSy has been associated with various metabolic pathways, which encompass nitrogen fixation (Baier et al., 2007; Kolman et al., 2015) and maintenance of mycorrhizae. For example, inhibition of nitrogen fixation in a *rug4* pea mutant with reduced SuSy levels in seeds and nodules. The converse is also true, as SuSy is not induced in soybean if the nodule symbionts fail to fix nitrogen (Gordon et al., 1999; Wang et al., 2025a). During the early development phase, it is induced specifically in root cells that have mycorrhizal arbuscules and sometimes also in the adjacent cells (Blee and Anderson, 2002; Kosová et al., 2023). *Sus1* from both *Zea mays* (Hardin et al., 2004) and *Glycine max* (Komina et al., 2002) binds to early nodulin 40 (ENOD40) peptides, which function as hormone-like peptides in the formation of root nodules in legumes (Zheng et al., 2011). A monomeric form of SuSy was identified as the nodulin-100 protein, which accumulates in soybean nodules (Zhang et al., 1999; Wienkoop et al., 2008). Therefore, the investigation of Susy genes in plants is pivotal for comprehending the complexities of plant physiology (Lu et al., 2022). The various roles of SuSy have been summarized in **Figure 2**.

### 3.1 Role of sucrose synthase in starch synthesis

**Figure 3:**
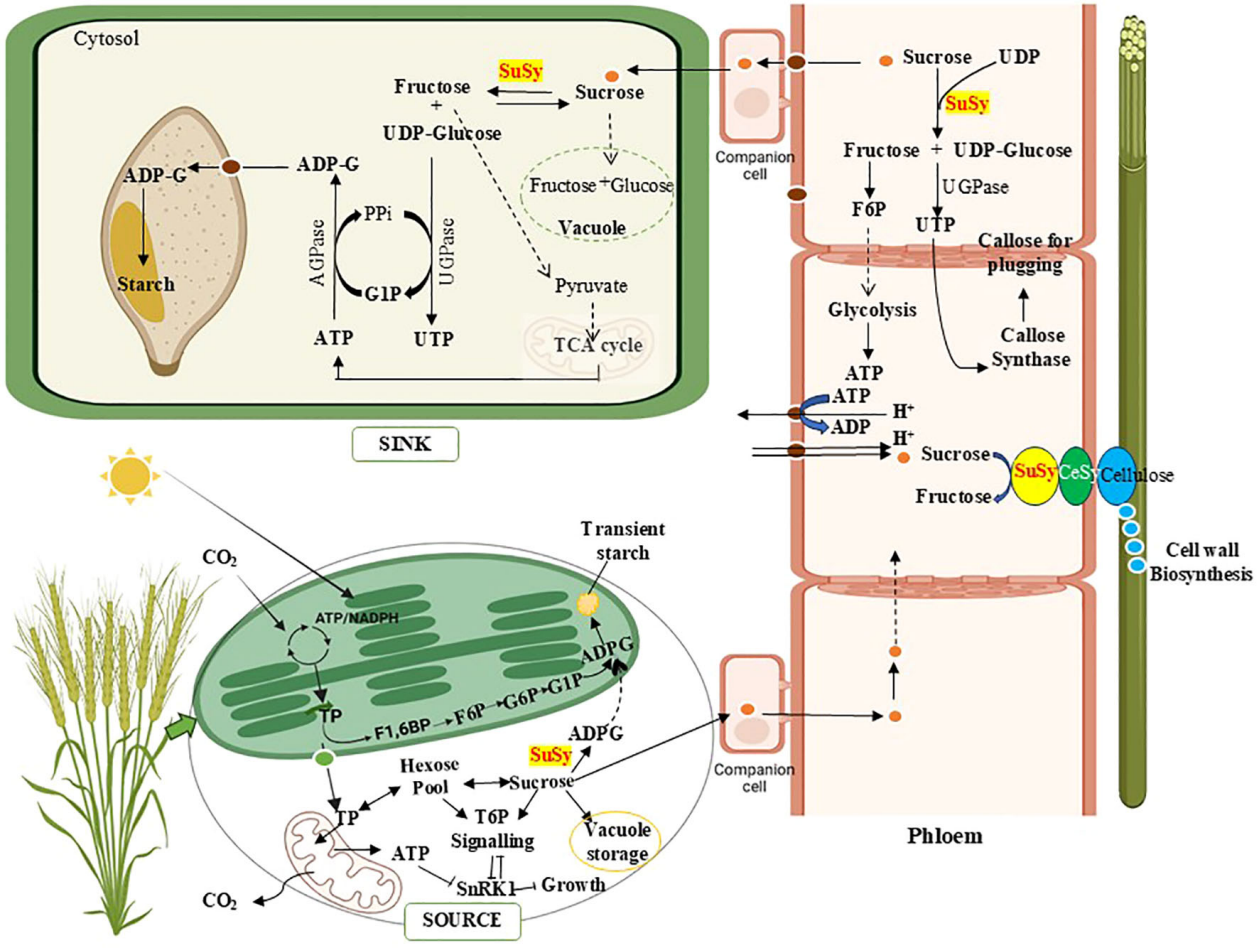
A simplified overview of sucrose synthase (SuSy) role in source-sink dynamics, cell wall and grain starch formation. SuSy facilitates the production of ADP-glucose (ADPG), connecting sucrose metabolism and starch synthesis in leaves. The enzyme’s activity channels UDP-glucose towards cellulose formation via transient association with membrane-bound cellulose synthase (CeSy), allowing efficient recycling of UDP (Uridine Diphosphate). In the phloem, SuSy supports callose deposition during protective plugging, while its reaction products contribute to ATP generation, maintaining sucrose gradients essential for transport. During grain development, SuSy breaks down sucrose into UDP-glucose for starch synthesis and fructose for pyruvate and ATP (Adenosine Tri-phosphate) production, coordinating energy supply for grain filling. Besides the plastid-localized isoform present in many plant tissues, cereals also contain a cytosolic version of ADP-Glucose-pyrophosphorylase (AGPase). SuSy also facilitates Triose 6 Phosphate (T6P) signaling which has an important role in plant growth and development. (Other enzymes involved in starch biosynthesis are not shown as they are beyond the scope of this review). G6P, Glucose 6 Phosphate; G1P, Glucose 1 Phosphate; F6P, Fructose 6 Phosphate; F1,6BP, Fructose 1,6 Bisphosphate; TP, Triose Phosphate; SnRK1, Sucrose non-fermenting 1- related protein kinase 1; UTP, Uridine Tri-phosphate; TCA, Tricarboxylic Acid Cycle. Created in BioRender.

In most higher plants, starch occurs in two primary forms: storage starch, which accumulates in amyloplasts as a long-term energy reserve, and transient starch, which is synthesized and degraded in chloroplasts of photosynthetic tissues in accordance with the diurnal light-dark cycle (Lloyd and Kossmann, 2015). In the leaves of crop plants, starch is synthesized from sucrose via two distinct pathways (Bahaji et al., 2014; Griffiths et al., 2016). In the first pathway, fructose-6-phosphate (F6P), generated from triose phosphates (TP), a key intermediate of the Calvin cycle, is converted to glucose-6-phosphate (G6P) inside the plastid, and this G6P serves as a substrate for starch biosynthesis. In the second pathway, TP is exported from the plastid to the cytosol, where it is converted into G6P or sucrose and subsequently transported back into the plastid for starch production (**Figure 3**). The role of SuSy comes in the latter one, in which sucrose is catabolized to ADP-G, which then re-enters the chloroplast to form starch, which is responsible for preventing carbon starvation during the night (Arias et al., 2014; MacNeill et al, 2017). This transitory starch biosynthesis model links the sucrose and starch metabolic pathways through the involvement of SuSy, which operates when cytosolic sucrose temporarily accumulates under light conditions and an ADP-glucose translocator is situated in the chloroplast envelope membranes. The controversy surrounding this has emerged, as a study conducted on Arabidopsis challenged the previous consensus by negating the role of SuSy and concluding ADPG pyrophosphorylase to be synthesizing the starch in chloroplasts (Baroja-Fernández et al., 2003; Crevillén et al., 2003; Fünfgeld et al., 2022). In most plant species, ADPG and starch biosynthesis typically occur within chloroplasts in photosynthetic tissues and in amyloplasts in heterotrophic organs. However, an exception to this pattern is observed in the endosperm of cereals and other members of the Poaceae family (Tetlow and Emes, 2017) Starch formation in wheat grains requires the sugar import, primarily in the form of sucrose transported from source tissues, a process facilitated by sucrose transporter (SUT) proteins (Aoki et al., 2004; Deol et al., 2013). Within the grains, SuSy plays a crucial role in starch biosynthesis by converting sucrose into UDPG, which is subsequently transformed into ADPG, the direct precursor for starch formation (Neuhaus and Emes, 2000; Hayashi et al., 2017). In cereal crops, a significant portion of ADPG is produced in the cytosol by AGPase and then transported into the amyloplasts through specific ADPG transporters, likely in exchange for AMP (Rösti et al., 2006; Figueroa et al., 2022).

The formation of storage starch in seeds, which serves as a carbon reserve for the next generation, depends on the availability of carbon in maternal tissues. Starch stored in leaves and other source organs, such as developing buds, flowers, siliques, and embryos, is remobilized to support reproductive development (Carlson et al., 2002; Lee et al., 2021). Studies favoring this hypothesis were found in potato and Arabidopsis leaves in which SuSy was found to be significantly exceeding its minimum activity required for starch accumulation during photosynthesis (Baroja-Fernández et al., 2012). Recent studies indicate that transient starch can also accumulate in storage plastids of non-photosynthetic cells, providing temporary carbon reserves for quick mobilization. This has been observed in the parenchyma of wheat peduncles (Scofield et al., 2009; Martínez-Peña et al., 2023) and in the floral organs of Arabidopsis during flower development (Hedhly et al., 2016). Even stomatal guard cells can function heterotrophically, relying primarily on sugars imported from the leaf mesophyll for starch synthesis, when excess sucrose is accumulated during stress (Dang et al., 2024). There is also an ongoing debate between SuSy and INV activity, as different studies offer varying conclusions that require further verification. During potato tuber development, starch accumulation is associated with an increase in SuSy activity and a decrease in acid INV activity (Appeldoorn et al., 1999), with the latter being regulated post-translationally by a proteinaceous inhibitor (Bracho and Whitaker, 1990). RNA profiling studies have suggested an inverse relationship between SuSy and acid INV expression (Zrenner et al., 1995; Zhang et al., 2024). It seems that the SuSy and acid INV-mediated sucrose cleavage pathways may be co-regulated in response to similar signals, and the balance between these pathways plays a key role in determining starch accumulation. A high-starch and low-starch phenotype were observed in SuSy-overexpressing and SuSy-antisensed potato tubers, respectively (Baroja-Fernández et al., 2009). Similarly, transgenic maize expressing expression of *ZmSUS1* has been associated with greater amylose content and larger seed size, accompanied by higher ADPG levels (Li et al., 2023). Research on wheat endosperm development reveals that the expression of cytoplasmic INVs does not align with starch biosynthesis gene expression. In contrast, SuSy showed a consistent expression pattern with starch accumulation, suggesting that sucrose is primarily processed by SuSy rather than cytoplasmic INVs during starch biosynthesis (Gu et al., 2021).

### 3.2 Role in sink-source dynamics

When sugar concentration increases in photosynthetic tissues, genes related to sucrose formation and amino acid synthesis in source tissues are upregulated, facilitating sugar translocation to sink tissues, which is essential for grain yield in crops like wheat (Aluko et al., 2021). Sucrose metabolism varies among different photosynthetic organs, potentially triggering molecular changes (Koch, 2004; Molero and Reynolds, 2020). Efforts to enhance sucrose-starch conversion have focused on increasing SuSy activity, as this sucrolytic enzyme plays a key role in determining sink strength and starch accumulation in both monocotyledonous and dicotyledonous plants (Jhandai et al., 2022). SuSy consistently exhibits higher activity than AGPase during seed development, aligning with ADPG and starch accumulation, further underscoring its importance in enhancing sink strength. Different SuSy genes exhibit diverse expression profiles and functions during the development of sink organs. SuSy is thus regarded as a reliable biochemical marker for sink strength and carbon allocation efficiency in plants, particularly those derived from SUS I (Xu et al., 2019). Generally, high SuSy activity is exhibited by source tissues as compared to sink tissues. However, increased biomass production and/or sucrose content was reported in switchgrass, tobacco, cotton, poplar, and rice due to overexpression of SuSy (Coleman et al., 2009; Jiang et al., 2012; Poovaiah et al., 2015; Fan et al., 2019). Additionally, plants with reduced SuSy expression showed noticeable changes in their phenotype (Craig et al., 1999; Ruan et al., 2003). Consistent with this proposition, Dehigaspitiya et al. (2021) found increased expression of *Sus1* in the pericarps of the three wheat genotypes during the grain enlargement stage, an interval marked by peak carbon assimilation and rapid biomass accumulation. This stage is characterized by heightened sink activity in wheat grains, including intense starch and amino acid synthesis. To meet the metabolic demands of these processes, increased sucrose cleavage is necessary, which aligns with the observed upregulation of *Sus1* in the pericarp at this phase. The resulting cleavage products are subsequently transported from the pericarp to the developing grain.

### 3.3 Sucrose synthase and sugar signals

Sucrose-cleaving enzymes can influence plant development by generating sugar signals. Beyond providing metabolic intermediates, the specific location and pathway of sucrose breakdown can produce unique sugar signaling patterns, which can significantly impact developmental processes (Mehdi et al., 2024). Starch is consequential for plant carbon metabolism and is, in fact, osmotically inert. Therefore, soluble sugars can serve as signals for carbon status, allowing plants to sense energy availability and integrate this information into developmental decision-making. Sucrose acts as an osmolyte in stomatal movement (Flütsch and Santelia, 2021) and as a metabolic substrate and signaling molecule, linking transpiration with sugar production and utilization. Sugar-induced stomatal closure appears to be evolutionarily conserved and biologically significant (Granot and Kelly, 2019). Increased sucrose cleavage enhances stomatal aperture. Guard cells in tobacco and Arabidopsis show higher SuSy activity than whole leaves, indicating its key role in sucrose metabolism (Bates et al., 2012). Overexpression of *SUS3* in guard cells of transgenic tobacco increased SuSy activity, stomatal aperture, conductance, transpiration, photosynthesis, and overall growth (Daloso et al., 2016; Piro et al., 2023). Within the entire plant system, hexose sugars encourage cellular proliferation and growth, whereas sucrose is more closely associated with cell specialization and tissue maturation. These observations, together with evidence from various plant systems, have contributed to the development of the INV/SuSy control hypothesis, which proposes a regulatory framework for key developmental transitions (Cosgrove, 1997). According to this concept, INV plays a crucial role in initiating and promoting the expansion of new sink organs, with vacuolar invertase activity often occurring before cell wall invertase becomes active. The function of cwINV frequently aligns with increased expression of hexose transporter genes in certain contexts (Feng et al., 2021). As development progresses toward storage and maturation phases, this shift is reflected in changes to the hexose-to-sucrose ratio (or the cell’s overall ‘sugar status’) and a switch from invertase-dominated to SuSy-mediated pathways for sucrose breakdown. During the early stages of seed development, INV and hexose transporters are more actively expressed (Weschke et al., 2003), while SuSy becomes essential for starch biosynthesis in the later grain-filling stages (Chen and Chourey, 1989; Yu et al., 2022). However, in certain localized regions, high levels of cwINV can remain active throughout maturation (Smeekens and Rook, 1997). These developmental processes are likely influenced by SuSy’s ability to modulate hexose-based sugar signals, especially at times when these signals might negatively affect differentiation or maturation (Lee et al., 2021). Overall, a wide range of evidence indicates that the relative activities of INV and SuSy can modulate plant development by differentially shaping sugar signaling pathways. Moreover, the interaction between sucrose cleavage products and hormone signaling, along with the hormonal regulation of sucrose metabolism itself, provides a mechanism for coordinating responses at the cellular level with the overall functioning of the whole plant. Simple sugars, such as sucrose and glucose, are potent inducers of SuSy gene expression (Quick and Schaffer, 2017). Studies in maize and rice reveal distinct responses among SuSy isomeric genes: while *Sus-1* enzyme levels increased tenfold in response to high carbohydrate concentrations, *Sh-1* did not show this increase, as sucrose can act as a repressor for it (Karrer and Rodriguez, 1992). The promoter region of SuSy, like other sucrose-inducible genes, contains a specific sucrose response element that promotes gene transcription through a yet-unknown mechanism. A wide range of sugar-responsive genes have been discovered, with their encoded proteins playing roles in various processes such as plant metabolism, light sensing, and regulation of the cell cycle (Lalonde et al., 1999; Dahiya et al., 2017). In several plant species, the genes for SuSy and INV are subjected to sugar regulation. During the developmental process in maize, SuSy and acid INV genes exhibit differential expression patterns. Genes activated by sugars, such as *Sus1* and *Ivr2*, are mainly expressed in tissues that import carbohydrates, while genes that are repressed by sugars and induced under starvation conditions, like *Sh1* and *Ivr1*, show increased expression particularly during reproductive stages. These expression patterns shift in response to changes in assimilate allocation, indicating a coordinated regulation of genes that respond differently to fluctuations in carbohydrate supply (reflecting ‘feast or famine’ scenarios) and developmental cues (Xu et al., 1996).

### 3.4 Role of sucrose synthase in abiotic stress tolerance

There is convincing proof that sucrose and starch metabolism are amongst the major regulatory systems granting resistance to abiotic stress, with UDPG playing a prominent role (Bala et al., 2010; Li et al., 2022). Decreases in non-reducing sugars and starch in cereal grains under stress suggest that more carbohydrates are being used to cope with stress. The sucrose metabolism may improve resistance to water deficit, but the relative contributions of these substances may differ depending on the genotype and the growth stage (Kumari and Asthir, 2016). Sucrose plays a critical role in conferring water stress tolerance under aerobic conditions in rice, with enhanced SuSy expression observed in both leaves and grains (Cheng et al., 2005; Fukuda et al., 2008). Reduction in grain and leaf starch content in some rice varieties indicates that sucrose metabolizing enzymes got significantly disrupted in them at all stages of plant growth (Kumari and Asthir, 2016). While those that showed an increased activity of SuSy in relation to protein and amino acid content coped better under stress conditions (Ruan, 2014). This upregulation suggests a coordinated downregulation of sucrose utilization pathways, promoting sucrose retention during stress. Under optimal oxygen conditions, SuSy expression is typically lower, but under severe stress, altered oxygen availability modulates sucrose metabolism through the regulation of SuSy activity. In hypoxic conditions, such as those encountered during seed germination under flooding, SuSy facilitates sucrose cleavage to support glycolysis and seedling growth by supplying intermediates (Yao et al., 2020). Unlike INVs, SuSy functions efficiently under oxygen-deficient conditions, conserving adenylate energy by generating UDPG instead of hexoses, which require ATP for subsequent glycolytic steps (Bologa et al., 2003; Tetlow and Emes, 2017). Under such situations, SuSy responds to cytosolic calcium spikes, supporting the biosynthesis of essential compounds like cellulose and callose (Schneider et al., 2016). During anaerobic stress in plants, a metabolic shift occurs from aerobic respiration to fermentation to sustain energy production. This shift involves increased glycolysis, driven in part by elevated sucrose cleavage via SuSy. For example, in maize seedlings, *Sh1* mRNA levels rise under prolonged anoxia, and *Sus1* quickly responds to hypoxia, boosting SuSy enzyme activity during long-term stress. Similarly, in cucumber, hypoxia stress from flooding induces *CsSUS3* expression and increases soluble SuSy activity, especially in lateral roots (Wang et al., 2014). In rice and Arabidopsis, the expression of *Sus1*, *AtSUS1*, and *AtSUS4*, respectively, is also enhanced under anaerobic conditions (Bieniawska et al., 2007).

Environmental stress can suppress photosynthesis, reducing sugar supply to sink tissues and impacting phloem function by disrupting callose deposition, which may in turn hinder sugar transport (Ceusters et al., 2016). Although excess sugars can protect membranes and proteins from stresses like cold, drought, salinity, and heat, they become largely unavailable for growth and can ultimately inhibit photosynthesis, limiting further sugar production. The balance between synthesis and breakdown of sugars, modulated by various enzymes, appears to influence sugar accumulation, as seen in rice and other cereals (Saeedipour, 2011; Kumari and Asthir, 2016). The involvement of sugars in enhancing abiotic stress tolerance is well established (Amist and Singh, 2020). However, several studies report that elevated starch biosynthesis under stress conditions provides carbon skeletons for the synthesis of compatible solutes, aiding plants in coping with such stresses (Thalmann and Santelia, 2017; Dong and Beckles, 2019). In many plants, starch metabolism serves as a crucial link between carbohydrate availability in source tissues and its allocation to sinks. Stress-induced shifts in carbon allocation may facilitate selective starch metabolism, as seen in cereals where stored carbon is redirected to essential functions or may bypass reproduction to prioritize survival during unfavorable conditions (Sulpice et al., 2009; Dong and Beckles, 2019). Efficient management of starch metabolism can enhance carbon use efficiency and help mitigate the negative impacts of stress. This is achieved through the selective or stepwise breakdown of starch in source tissues, sink tissues, or both. For instance, in sink organs, converting sugars into starch helps maintain low local sugar concentrations, which promotes a continued flow of assimilates from source tissues, where sugar levels remain high. Whether starch in a given tissue functions as a ‘sugar reservoir’ or a ‘sugar consumer’ during stress adaptation depends partly on the plant’s developmental phase, which is influenced by hormonal signaling pathways (Xiao-Li et al., 2022). In heterotrophic tissues such as roots, tubers, and seed endosperms, sucrose acts as a critical energy supply for metabolic processes and also contributes to the stabilization of cellular membranes and proteins under abiotic stress conditions (Sulpice et al., 2009; Julius et al., 2017; Durand et al., 2018). This SuSy-driven starch synthesis supports energy storage and resilience under stress (Figueroa et al., 2013; Saddhe et al., 2021). The semi-crystalline nature of starch makes it water-insoluble and osmotically inactive (Goren et al., 2018). By storing carbohydrates in a water-insoluble form, plants prevent excessive water uptake that could otherwise disrupt cellular function under stress. Typically, cereal endosperm starch adopts an A-type allomorph, characterized by densely packed, shorter glucan chains that repel water (Hsein-Chih and Sarko, 1978; Imberty et al., 1988), making it less capable of water absorption. This structure supports energy storage without increasing water content, which is advantageous for stress tolerance, especially in dry or low-water conditions. In contrast, transient starch in leaves and tubers usually exhibits the B-type allomorph with loosely packed, longer α-glucan chains that can absorb more water (Imberty and Pérez, 1988). This structure helps buffer against sudden water fluctuations, which can be beneficial for stress avoidance by maintaining water balance during temporary water availability changes.

Adaptive responses in stress-tolerant lines may involve accelerated starch-to-sugar conversion to prevent sugar depletion without inducing sugar injury and reducing sink strength (Dong and Beckles, 2019). However, under mild water deficit, enzymes in the sucrose-to-starch pathway, including SuSy, are often upregulated in cereal grains, contributing to enhanced starch reserves (Luo et al., 2021). Water deficits increase SuSy activity in both drought-sensitive and resistant rice varieties, suggesting SuSy’s role in osmotic adjustment (Wang et al., 2025b). In drought-resistant varieties, higher starch content around root vascular tissues suggests an adaptive response to water stress (Singh et al., 2013). In maize, overexpression of *ZmSUS1* in maize kernels, leaves, and roots enhances drought resistance by modulating sucrose metabolism and increasing soluble sugar content, which helps maintain cellular osmotic balance and energy under stress. In drought-adapted plants like Arabidopsis, mutants with reduced guard cell sugar content show limited stomatal opening, enhancing drought tolerance by conserving water (Prasch et al., 2015). The *Sus3* isoform has been specifically identified in the guard cells of Arabidopsis (Bieniawska et al., 2007) and has also been detected in potato, particularly under drought stress conditions (Kopka et al., 1997). Studies indicate that SuSy isoforms are regulated by distinct mechanisms that depend on sugar concentrations, as observed in maize (Koch et al., 1992) and citrus (Komatsu et al., 2002). They showed higher SuSy activity, relative water content, proline, and abscisic acid levels in leaves (Xiao et al., 2024). Notably, several studies have highlighted the involvement of specific SuSy isoforms in cold stress tolerance. For instance, upregulation of *SUS3* in tomato (Li et al., 2024), *BjuSUS08* in mustard (Brassica juncea) (Li et al., 2021), and *HbSus5* in roots and leaves of *Hevea brasiliensis* on exposure to low temperature (Xiao et al., 2014). In mustard, however, *BjuSUS03* is significantly upregulated under multiple abiotic stresses (Li et al., 2021). In sorghum, *SbSusy1*, *SbSusy3*, *SbSusy4*, and *SbSusy5* are induced at the seedling stage under drought and salt stress, but their expression is suppressed under high osmotic pressure (Lu et al., 2022). Collectively, these results underscore the functional diversity of SuSy isoforms in mediating plant responses to various abiotic stresses.

## 4 Role of sucrose synthase in heat stress tolerance

Heat stress impacts various cellular and physiological processes, including cell growth and macromolecule interactions. With rising global temperatures, understanding these effects is crucial for selecting plants better suited to a changing climate (Teixeira et al., 2013). According to the IPCC’s synthesis report, global temperatures are expected to surpass 1.5°C between 2021 and 2040 (Lee et al., 2024). Heat stress refers to temperatures that surpass critical limits, negatively impacting crop yield and quality. In bread wheat (*Triticum aestivum*), temperatures rising above the optimal range of 17–25°C, particularly daytime temperatures exceeding 32°C during grain filling, can induce stress responses that compromise both yield and grain quality (Frieler et al., 2017; Telfer et al., 2018; Zampieri et al., 2018). The reduced yield is due to disruptions in floret initiation, including floral deformities like pistil overdevelopment and stamen underdevelopment, as well as decreased pollen viability. SuSy has been identified as playing a role in plant responses to heat stress.

### 4.1 Reproductive development and pollen viability under heat stress

Pollen formation is a temperature-sensitive developmental stage, and key stages like microsporogenesis and microgametogenesis can be disrupted if temperature rises above threshold, especially in cereals (Chaturvedi et al., 2021; Shi et al., 2022). A temperature above 30°C during meiosis till pollen maturation negatively impacts pollen viability, reducing fertilization and seed production (Ullah et al., 2022). In maize, heat stress before or during tassel emergence can cause tassel desiccation and death, as well as reduced pollen production (McNellie et al., 2018). Limited photo assimilate availability in source leaves and sensitivity to photoperiod changes hinder floret formation and grain development under heat stress, emphasizing the need for sufficient assimilate reserves in vegetative tissues (Aiqing et al., 2018; Jagadish, 2020). The maize transcriptomic analysis showed a correlation between reduced pollen viability and significant reductions in *Sh-1* and *sus1* gene expression under heat stress as compared to control conditions (Li et al., 2022). These reductions likely caused lower UDPG and higher sucrose levels (Kaur et al., 2019). Heat-tolerant tomato cultivars, unlike heat-susceptible genotypes, sustain pollen starch content and reduced sucrose metabolism under stress, which supports improved fertility (Nepi et al., 2001; Firon et al., 2006; Kumar et al., 2015). Enhanced SuSy activity has been observed in the anthers of heat-tolerant tomato, alfalfa (Mo et al., 2011), and rice (Guan et al., 2023). Although some studies have reported no significant changes in tomato and potato under similar conditions (Lorenzen and Lafta, 1996; Li et al., 2012). However, a decline or impairment in INV activity was observed, with development proceeding via the SuSy-mediated pathway, which is considered more energy-efficient (Guan et al., 2023). Enzymatic assays also verified that SuSy activity is positively related to pollen viability, highlighting the importance of carbohydrate metabolism in sustaining pollen function (Begcy et al., 2019; Jagadish, 2020). Sucrose restriction to female reproductive parts also hinders pollen tube growth as observed in cotton (Hu et al., 2019) and maize (Wang et al., 2023). In gametophytes like pollen grains and ovaries, starch biosynthesis initially boosts sink capacity but later breaks down to release sugars, providing energy for growth—a key factor for reproductive success.

**Figure 4:**
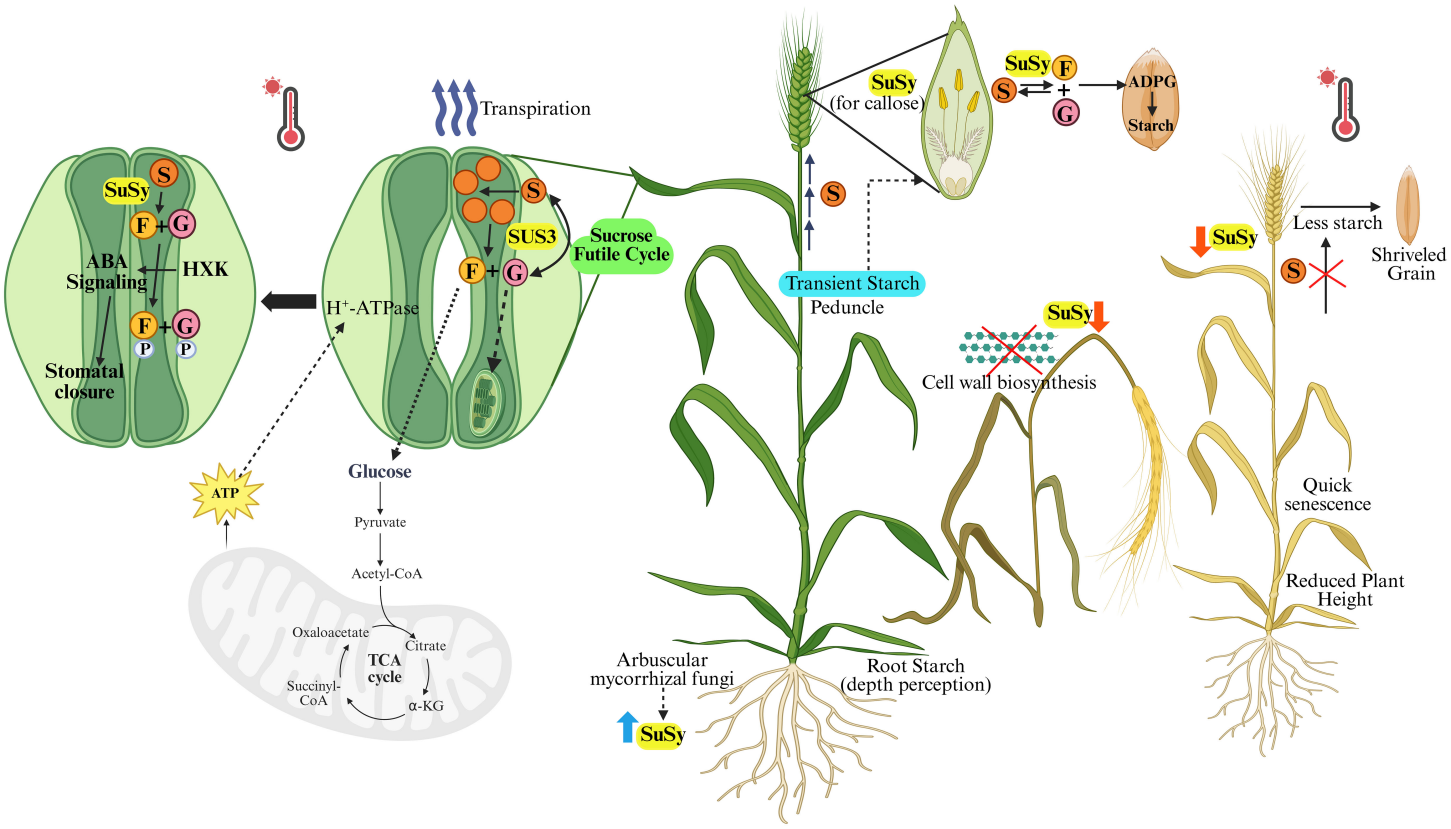
Sucrose synthase (SuSy) exhibits dynamic localization depending on the metabolic environment and plays a critical role in cereal crops’ response to heat stress (HS). During HS, stomatal closure reduces water loss, a process influenced by SuSy through sucrose degradation in guard cells into UDP-glucose and fructose. These sugars are phosphorylated by hexokinase, triggering ABA signaling that leads to stomatal closure. While increased sucrose concentration provides osmotic protection, prolonged accumulation can damage photosynthesis. SuSy regulates a sucrose futile cycle to mitigate this stress. Heat stress impairs SuSy activity, disrupting cell wall biosynthesis, growth, and floral development, ultimately compromising grain formation in a heat susceptible genotype. Additionally, SuSy facilitates the remobilization of transient starch from tissues like the peduncle to sink organs and the endosperm for starch synthesis under HS. Root architecture and water use efficiency, supported by arbuscular mycorrhizal fungi, further influence SuSy activity and help plants cope with heat stress. (S, Sucrose; G, UDP-Glucose; F, Fructose; P, Phosphate; SUS3, SuSy isoform; HXK, Hexokinase; ABA, Abscisic Acid) Created in BioRender.

SuSy abundance, distribution, and functionality depend on cytoskeleton and membrane activity (Cai et al., 2011). Heat shock may alter cytoskeleton integrity, impacting proteins reliant on cytoskeleton dynamics for localization, including those that interact directly with it. Heat stress can thus disrupt SuSy localization in the cell wall, as seen in pollen tubes post-heat shock, where it redistributes differentially (Parrotta et al., 2016). SuSy is vital in pollen tubes, as it is required for cellulose and callose synthesis. Disruption in it can therefore jeopardize the plant’s growth and development, potentially preventing the plant from reaching maturity or producing a viable harvest (**Figure 4**). Heat stress may redirect SuSy within the cell, reducing its role in cell wall synthesis to conserve energy. To rebalance carbohydrate content, SuSy may either be redirected to the cytoplasm or remain inactive within the endomembrane system, affecting the enzyme’s association with actin and its location in the cell (Hardin et al., 2004; Duncan and Huber, 2007). Vesicle delivery disruptions might also impact SuSy’s distribution and, consequently, cell wall synthesis. This suggests that altering SuSy distribution may help pollen tubes adapt to environmental changes caused by the inhibition of metabolic pathways in heat-stressed pollen grains.

### 4.2 Sucrose synthase activity in guard cell signaling pathway

*SUS3* plays a pivotal role in guard cell metabolism, especially towards the end of the day when sugar levels are high. It is thought to participate in a proposed sucrose futile cycle (**Figure 4**), where continuous flux of sucrose synthesis and degradation occur simultaneously, balancing cytosolic sugar levels and preventing excess starch formation (Daloso et al., 2016). Rather than contributing significantly to starch, most *SUS3*-derived metabolites are likely funneled into the tricarboxylic acid (TCA) cycle and oxidative phosphorylation, generating ATP and organic acids essential for respiration (**Figure 4**). Experimental reduction of *SUS3* expression in guard cells led to decreased CO_2_ assimilation and transpiration, underscoring its importance in regulating stomatal function and overall plant productivity (Antunes et al., 2017; Piro et al., 2023). Enhancing *SUS3* activity could thus represent a promising strategy for improving water-use efficiency and growth. Disruption of the sucrose futile cycle, such as in *sus3* mutants, may lead to starch overaccumulation by redirecting intermediates like G1P into plastids via G1PT transporters. More broadly, starch accumulation is tightly linked to metabolite homeostasis, with key regulators including G6P, F6P, UDPG, and sucrose. G6P levels, which influence both starch synthesis and degradation, are modulated by sucrose metabolism and transport across the amyloplast (Getz and Klein, 1994; Nguyen-Quoc and Foyer, 2001). Water use efficiency can also be increased by the activity of arbuscular mycorrhizal fungi, which is linked with abiotic stress tolerance, as suggested by a study on bread wheat (Bernardo et al., 2019). SuSy, along with other enzymes involved in cell wall remodeling, is modulated by these fungi as part of their regulation of carbohydrate metabolism, cytoskeleton dynamics, and stress- or defense-related proteins (Kosová et al., 2023). Thus, SuSy contributes to stress tolerance through complex and diverse mechanisms, underscoring its potential for further exploration.

### 4.3 Source-sink remobilization and grain filling under heat stress

High night temperatures led to increased proteinogenic amino acids and sugars like sucrose and raffinose in winter wheat spikes (Impa et al., 2019), supporting cell division but hindering starch accumulation in early seed stages (Weber et al., 1997). The formation of endosperm cells occurs during the early seed-filling stage, and their number and final size are governed by the rate and duration of seed filling, both of which are negatively impacted by high temperatures (Nicolas et al., 1985; Dong et al., 2021). Heat stress reduces the levels of total non-structural carbohydrates (NSC), impacting the balance between soluble sugars and starch (Kumar et al., 2023). Heat-tolerant wheat varieties transported NSC from stems and leaves to the kernel more efficiently than susceptible varieties, maximizing grain yield and securing reserves for seedling germination (Kumar et al., 2017). Similarly, heat-tolerant rice cultivars exhibited lower NSC content in the stem culm due to enhanced remobilization to the grain under high temperatures (Tanamachi et al., 2016). During late seed development, starch synthesis in the endosperm suffers due to limited assimilate supply or disruptions in starch biosynthesis, which leads to loosely packed starch grains (Barnabás et al., 2008). Paradoxically, a brief initial exposure to high temperatures can temporarily boost starch levels in cereal grains, as observed in barley (Wallwork et al., 1998), rice (Bahuguna et al., 2017), and wheat (Nicolas et al., 1985; Dong and Beckles, 2019). In barley this increase is linked to heightened activity of starch biosynthetic enzymes, including SuSy (Wallwork et al., 1998). However, prolonged heat stress can alter the structure of storage products in barley and disrupt starch accumulation in wheat (Ullah et al., 2022) and rice (Zhang et al., 2018) by decreasing the transcription and activity of essential starch biosynthetic enzymes, which raises the sugar content within the grains (Dong and Beckles, 2019). In rice an accelerated endosperm development under heat stress leads to the formation of chalky grains (Wada et al., 2019). A study by Takehara et al. (2018) found that *OsSus3*, which is highly expressed during seed ripening, may help protect against the chalky grain phenotype in brown rice caused by heat stress. The gene underlying the QTL *Apq1* (Appearance quality of brown rice 1) is the thermos-responsive *Sus3* allele, whose increased expression during ripening enhances rice tolerance to high temperatures. Post-anthesis heat stress significantly reduces the grain-filling duration and disrupts assimilate allocation, resulting in yield losses ranging from 6–51% under controlled conditions and 2–27% under field conditions in wheat (Bergkamp et al., 2018). A shorter grain-filling period often results from limited sucrose supply to the developing kernels and decreased activity of key enzymes responsible for sugar and starch metabolism. Efficient transport of photoassimilates from source tissues like leaves, stems, and spikes to grains is essential for effective grain filling, highlighting the importance of optimizing source-sink relationships (Abdelrahman et al., 2020). Grain yields are significantly affected by both the availability of assimilates (source limitation) and the grain’s capacity to store them (sink limitation). Although some research minimizes the impact of source limitation under heat stress, it supports sink limitation, pointing to reductions in grain size and number as primary causes of yield decline (Kumar et al., 2017; Hütsch et al., 2019). Strengthening grains to assimilate storage capacity, particularly by enhancing starch synthesis enzyme activity, is vital for achieving optimal yield potential (Borrill et al., 2015 **).** While the negative impact of heat stress on yield is well-documented (Mendanha et al., 2018; Fan et al., 2018; Zhang et al., 2018), metabolic responses, especially in source-sink dynamics and yield, are less understood (Abdelrahman et al., 2020). The impact of heat stress on crop yield is influenced by plant genetics and physiological responses, which vary with the developmental stage and regulation of nitrogen and carbon fluxes (Sehgal et al., 2018).

The majority of studies have found that heat stress repressed SuSy activity and decreased sucrose levels in wheat grains (Asthir et al., 2012), especially in late-sown conditions (Dale and Housley, 1986). In thermo-sensitive lines, SuSy was disrupted in both the flag leaf (source) and spikes (sink), which resulted in low sugar content in the rachis but high levels in the spikelet. In the tolerant cultivar, the enzyme activity remained relatively high, with only a slight dip at the vegetative stage (Bahuguna et al., 2017). The fluctuations in SuSy activity are correlated with the changes in starch content in grain of both heat-susceptible and tolerant cultivars (Bala et al., 2010). Its activity in the synthesis direction peaks around 7 days after anthesis (DAA) under both normal and late sowing conditions, with a steady decline toward grain maturity (Zhao et al., 2008). The expression patterns of three SuSy genes were examined during grain filling, and it was found that *TaSuSy2* showed elevated expression during early to mid-filling. SuSy is critical in wheat endosperm development, showing peak activity during the rapid grain-filling phase (8–25 DAA) (Mukherjee et al., 2015). Histochemical assays reveal that SuSy in the endosperm shifts progressively from the apical to the basal region, aligning with the areas of starch synthesis during kernel development (Wittich and Vreugdenhil, 1998). Grains with greater water content and maximal dry weight also show elevated SuSy activity, indicating its role in determining kernel weight (Dale and Housley, 1986; Zi et al., 2018). This correlation suggests that SuSy contributes to enhanced carbohydrate partitioning and starch accumulation, impacting grain filling and final yield under varying growth conditions (**Figure 4**) (Sekhar et al., 2015; Berahim et al., 2019). Gene diversity analyses across wheat accessions have identified specific *TaSuSy1* and *TaSuSy2* haplotypes that are linked to variations in thousand-grain weight (Jiang et al., 2011; Hou et al., 2014). In durum wheat, *SuSy2* expression peaks during the seed’s milk stage, supporting its role in starch accumulation (Volpicella et al., 2016). Additionally, in barley, changes in starch accumulation under drought correlate with SNPs in *SuSy1* and *SuSy2* genes, indicating their role in sucrose hydrolysis during stress (Worch et al., 2011). In rice endosperm, SuSy activity is lower in inferior spikelets compared to superior ones and is positively linked to the grain’s ability to take up sucrose, thereby acting as a potential indicator of high grain yield (Counce and Gravois, 2006; Panda et al., 2015; Zhang et al., 2018). Increased SuSy activity has also been reported in heat-tolerant chickpea genotypes and sugarcane along with an elevated sucrose level, highlighting the importance of maintaining sucrose during stress (Kaushal et al., 2013; Noman et al., 2022; Shanthi et al., 2023). Heat-tolerant Agrostis grass accumulated SuSy, possibly to provide protective metabolites that contribute to enhanced root heat tolerance (Xu et al., 2008). In maize, heat stress led to downregulation of SuSy genes without impacting enzyme activity (Duke and Doehlert, 1996; Wilhelm et al., 1999). In contrast, recent studies indicated that an early stress exposure led to decreased SuSy activity in maize kernels during the filling stage (Liu et al., 2022). During grain development, SuSy predominates over INV, favoring pathways based on tissue-specific processes (Sung et al., 1994; Wang et al., 2023). In rapidly growing and storage tissues, SuSy-driven pathways are predominant, while acid INV activity is more prominent in expanding tissues. Sucrose-metabolizing enzymes (SPS, SuSy, and INV) function together to sustain growth by synthesizing sucrose and providing hexoses for development (Nguyen-Quoc and Foyer, 2001).

### 4.4 Contrasting carbohydrate storage strategies in crop plants

Species like tobacco, Arabidopsis, tomato, and potato are categorized as having ‘starch leaves,’ while wheat, rice, and barley are considered to have ‘sugar leaves’—a distinction based on the leaf starch-to-sugar ratio and the function of transitory starch (Cook et al., 2012; Okamura et al., 2014). Despite being classified as sugar-leaf plants, rice utilizes leaf starch primarily to enhance source capacity under high light conditions, such as full sunlight in paddy fields, rather than serving as a nighttime carbon reserve for growth (Okamura et al., 2017). Starch formation in the leaf also leads to transcriptional upregulation of stress-related genes (Xue et al., 2008). One potential strategy for enhancing starch production in heterotrophic organs involves the ectopic expression of SuSy in plastids, as seen in cyanobacteria. Cyanobacterial SuSys show strong affinity for ADP. Therefore, an innovative approach to boost starch content in crop plants where a significant portion of sucrose is localized within plastids could involve expressing cyanobacterial SuSy in plastids to generate ADPG (Curatti et al., 2000; Figueroa et al., 2013). The pathway for starch degradation in storage organs differs from that in leaves (Zeeman et al., 2007, 2010), with some starch turnover likely persisting throughout the development of storage tissues. This turnover may be more pronounced in organs that store transient starch, such as lotus embryos and tomato fruits, reflecting starch’s adaptive role across plant organs (Lloyd and Kossmann, 2015; Thalmann and Santelia, 2017).

In C3 cereals, ear photosynthesis plays a crucial role in supplying photoassimilates, particularly under unfavorable environmental conditions (Tambussi et al., 2007; Sanchez-Bragado et al., 2020). Stems and leaf sheaths serve as temporary carbon storage sites, which remobilize stored carbon to reproductive tissues and significantly aid grain filling during later growth stages (Mathan et al., 2021). In temperate cereals like wheat, fructans and sucrose are the primary storage carbohydrates, although some starch accumulates before anthesis (Schnyder et al., 1993; Scofield et al., 2009). This stored starch may support early reproductive organ development or peduncle growth. In wheat peduncles, one region functions as a starch source, while another serves as a sink (**Figure 3**) (Scofield et al., 2009; Ntawuguranayo et al., 2024). Unlike wheat, rice lacks fructan biosynthesis enzymes, relying instead on transient starch storage sites (Livingston et al., 2009). During vegetative growth, the rice stem acts as a sink, accumulating starch from leaf photosynthates. Post-head development, the stem shifts to a source, remobilizing starch for grain filling, contributing approximately 25% of rice grain carbohydrates (Perez et al., 1971; Hirose et al., 2014). These carbohydrate reserves in vegetative tissues are vital for reducing yield losses under stress conditions, especially during the grain-filling stage. In rice, high temperature led to an increase in SuSy activity during early developmental stages, whereas the activity decreased in middle to later stages (Chaturvedi et al., 2017). AGPase activity responded to temperature changes; however, its effect was less pronounced than that of SuSy activity (Kato et al., 2007). Heat-induced repression of SuSy protein may impair its catalytic function, reducing the production of precursor monomers essential for generating AGPase substrates, as observed in rice (Cheng et al., 2005) and barley (Macleod and Duffus, 1988).

### 4.5 Positioning SuSy amongst ROS, NO, ABA, and auxin signaling for plant heat stress response

**Figure 5:**
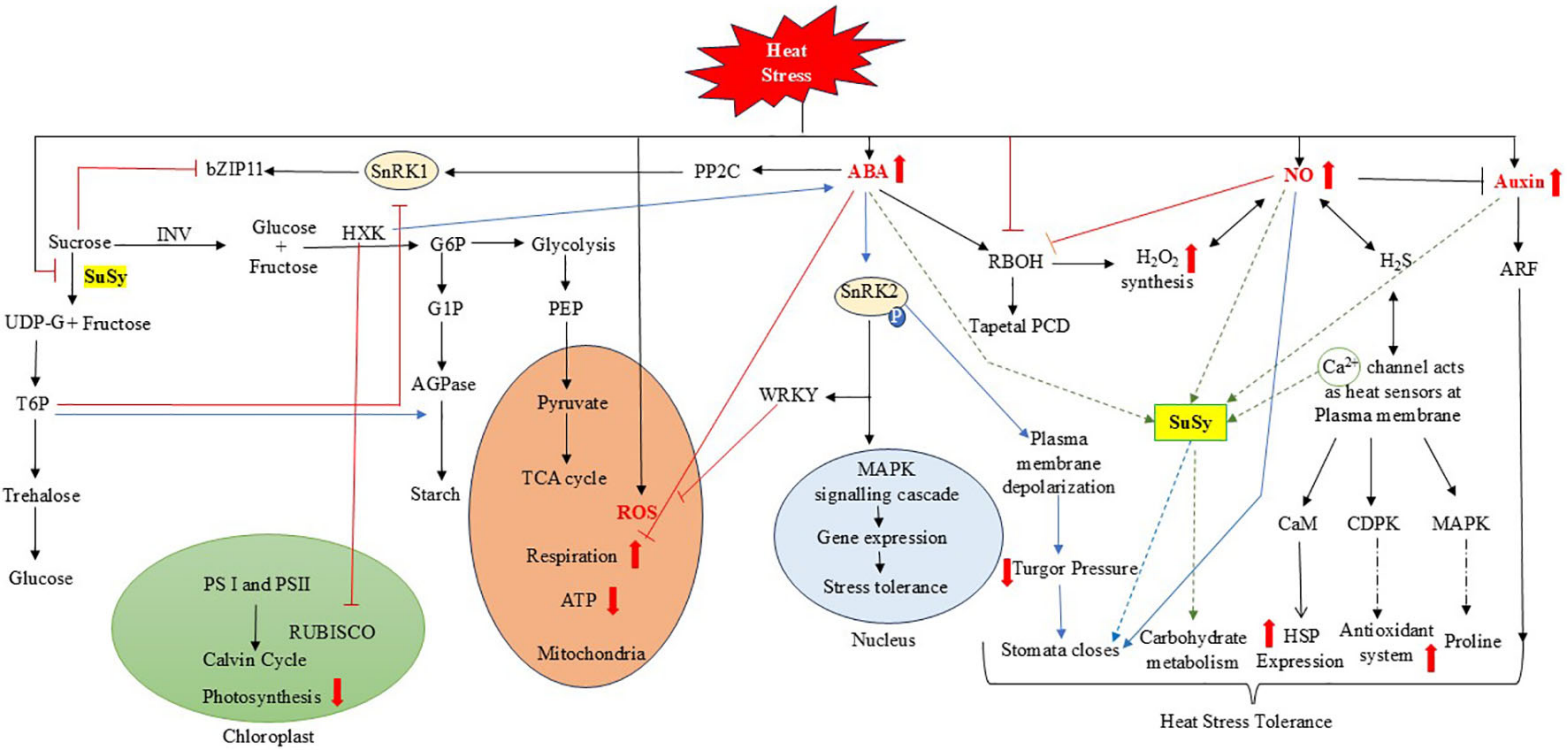
Schematic illustration of interactions among sucrose synthase (SuSy), abscisic acid (ABA), reactive oxygen species (ROS), nitric oxide (NO), and other signaling molecules in heat stress tolerance. Blue arrows indicate stomatal signaling; green arrows represent phytohormonal regulation of SuSy expression; red blunt-ended arrows denote inhibition; bold red arrows show increases/decreases; and black arrows represent activation or metabolic progression. HS triggers multiple physiological changes that are regulated by phytohormones (e.g., ABA, auxin) and signaling molecules (e.g., NO). Hexokinase (HXK) not only suppresses RUBISCO activity, leading to reduced photosynthesis, but also activates the ABA signaling pathway. The enhanced activity of these signaling components initiates cascades that induce stress-responsive gene expression and heat shock protein (HSP) synthesis. These responses are also associated with increased SuSy activity, highlighting its potential role in conferring HS tolerance. For a detailed description, see Section 4.5. INV, invertase; bZIP11, basic leucine zipper; SnRK1, sucrose non-fermenting 1-related protein kinase 1; PP2C, protein phosphatase 2C; T6P, triose-6-phosphate; PS I, photosystem I; PS II, photosystem II; RUBISCO, ribulose-1,5-bisphosphate carboxylase/oxygenase; PEP, phosphoenolpyruvate; TCA, tricarboxylic acid cycle; ATP, adenosine triphosphate; PCD, programmed cell death; H_2_O_2_, hydrogen peroxide; CaM, calmodulin; CDPK, calcium-dependent protein kinases; MAPK, mitogen-activated protein kinases; RBOH, respiratory burst oxidase homolog; Ca²^+^, calcium; HSP, heat shock protein; WRKY, transcription factor; ARF, Auxin responsive factors.

Plants employ a range of strategies to mitigate heat stress, including the activation of antioxidant defense systems, synthesis of heat shock proteins (HSPs), modulation of phytohormone levels, and regulation of sugar metabolism (**Figure 5**). Heat stress impairs photosynthesis by disrupting PSII efficiency, reducing Rubisco activity, and enhancing photorespiration. This leads to excessive generation of reactive oxygen species (ROS), which compromise cellular function and integrity (Dwivedi et al., 2017; Telfer et al., 2018). However, the role of ROS is complex, and increasing evidence suggests that they also function as critical signaling molecules. They can regulate plant development and stress-responsive gene expression (Mittler et al., 2022), including the activation of genes encoding antioxidant enzymes and those involved in hydrogen peroxide (H_2_O_2_) production (Suzuki et al., 2011; Xue et al., 2020). Nevertheless, excessive ROS accumulation under heat stress can be detrimental, affecting cell differentiation, root elongation, and stomatal behavior (Muhlemann et al., 2018). Stomatal closure is a well-known adaptive response to heat stress, aimed at reducing transpiration water loss. Abscisic acid (ABA) plays a key role in heat stress signaling as well, particularly in mediating stomatal responses. Another important signaling molecule, nitric oxide (NO), influences guard cell function by altering ion fluxes and water movement, thereby modulating turgor pressure and stomatal aperture (Lau et al., 2021). NO is thought to act downstream of ABA in this signaling cascade, reinforcing stomatal closure during thermal stress (Sun et al., 2019). While SuSy has a role in stomatal closure (discussed in *subsection 4.2*), the proposed way it interacts with ROS, NO, and ABA has been illustrated in **Figure 5**.

ROS and NO are key signaling molecules in pollen-pistil recognition and pollen tube directional growth. Elevated ROS and NO levels facilitate communication between pollen and stigma cells, while NO helps modulate ROS during pollen arrival. These processes are highly heat-sensitive; elevated temperatures can disrupt programmed cell death (PCD) needed for tapetal disruption for pollen development. Due to the high mitochondrial activity in pollen and tapetal cells, excess ROS under heat stress can lead to uncontrolled PCD and damage reproductive tissues. Thus, tight regulation of ROS is essential for supporting necessary PCD for fertilization while preventing heat-induced cellular damage. Several studies have shown that NO levels increase in response to elevated temperatures across various plant species and depend on the intensity and duration of heat exposure (Nabi et al., 2019). Exogenous NO application has been found to alleviate heat-induced cellular and oxidative damage in wheat callus cultures and plants (Bavita et al., 2012). Under heat stress, increased NO accumulation has been associated with elevated sucrose levels in leaves and anthers, indicating a potential protective role in preserving reproductive function (Choukri et al., 2022). NO also promotes the expression of key metabolic enzymes, including Rubisco and SuSy, as reported in heat-stressed lentils (Sita et al., 2021). Enhanced SuSy activity in NO-treated plants may help maintain cellular function in both leaves and reproductive tissues. Additionally, the accumulation of reducing sugars such as hexoses supports osmotic balance, energy supply, and structural carbohydrate synthesis under heat stress.

Studies have shown that auxin contributes to heat stress tolerance in cereals such as rice by safeguarding spikelet fertility and grain yield (Sharma et al., 2018; Chen et al., 2024) and in wheat as well (Abeysingha et al., 2021). Auxin and its signaling pathways modulate thermomorphogenic responses, enabling plants to balance growth and stress defense under elevated temperatures. Recent findings indicate that auxin also promotes starch accumulation (Ross and McAdam, 2025). Interestingly, suppression of SuSy has been linked to altered auxin signaling and changes in leaf morphology in tomato (Goren et al., 2017). Plant hormones, particularly ABA, serve as crucial internal signals in mediating heat stress responses during grain filling in wheat (Kumar et al., 2015). ABA has been shown to induce thermotolerance in both wheat and rice (Hu et al., 2018; Zhang et al., 2014). It enhances the expression of genes involved in the ascorbate–glutathione cycle, contributing to ROS detoxification. Under heat stress, ABA also upregulates sucrose transporters and metabolism-related genes such as SuSy and INV, supporting ATP production and maintaining energy balance (Chen et al., 2019). ABA contributes significantly to the grain filling process by enhancing starch accumulation efficiency. This effect is largely attributed to its regulation of SuSy activity (Tang et al., 2009). SuSy is a critical enzyme in grain filling, and its function is tightly regulated by both sucrose and ABA at the levels of enzyme activity and protein expression. Exogenous ABA application boosts SuSy activity and the expression of starch synthesis genes, improving carbohydrate content—including soluble sugars, starch, and NSCs. These changes contribute to better thermotolerance through enhanced HSP expression and antioxidant activity. ABA-mediated regulation of sugar metabolism has also been linked to improved spikelet protection under heat stress, suggesting a tightly coordinated system that supports reproductive resilience. SnRK1 is a key regulator of ABA signaling and is involved in stress adaptation (Tsai and Gazzarrini, 2014). It plays a pivotal role in coordinating plant responses to stress and regulating carbon signaling pathways, affecting the expression of thousands of genes in mesophyll cells (Baena González et al., 2007; Nunes et al., 2013). Its activity is specifically suppressed by G6P, G1P, and T6P, which tend to accumulate during active photosynthesis (Nunes et al., 2013). In potatoes, SnRK1 has been shown to enhance SuSy expression in developing tubers and leaves in response to sucrose availability (Purcell et al., 1998) and is also implicated in regulating starch breakdown via bZIP transcription factors and microRNAs (Confraria et al., 2013). Additionally, SnRK1 and hexokinase (HK) independently contribute to increased starch synthesis by activating AGPase through redox-based mechanisms when sucrose and glucose levels rise (McKibbin et al., 2006). Generally, genes upregulated by SnRK1 are downregulated by T6P. The inhibition of SnRK1 by T6P regulates sink tissue development rates by controlling SnRK1’s influence on vINV and SuSy (Martínez-Barajas et al., 2011; Lin et al., 2015). However, the interplay between SnRK1, T6P, and starch metabolism remains complex and not fully understood. Evidence suggests that long-distance signaling may coordinate responses between heat-exposed and non-exposed tissues, linking heat stress mechanisms with carbon transport between source and sink tissues (Suzuki and Katano, 2018). ROS-dependent signals are proposed to integrate with heat-induced long-distance signaling, potentially enhancing ABA synthesis while downregulating sugar metabolism intermediates in leaves not directly exposed to heat. Additionally, a transient rise in cytosolic Ca²^+^ levels is a well-established response to heat stress, and calcium signaling may converge with ABA and SnRK1 pathways (Wasilewska et al., 2008; Goswami et al., 2015). Bhatia and Asthir (2014) reported that SuSy activity increased with a Ca²^+^ spike at ambient temperature, and in certain wheat varieties, this enhancement was also observed under heat stress. This Ca²^+^ signaling has also been shown to interact with ROS-mediated systemic responses to localized abiotic stimuli (Fichman and Mittler, 2021). Microarray analyses have identified sugar-responsive elements in the promoters of several heat-inducible genes, suggesting that sugar signaling contributes to the development and maintenance of acquired thermotolerance. Together, these findings highlight the complex integration of ROS production, Ca²^+^ signaling, metabolite sensing, and hormonal regulation in balancing plant growth and thermotolerance. A detailed crosstalk among them is described in **Figure 5**.

## 5 Conclusion

Extensive research conducted over the past decades has unequivocally established the multifaceted contributions of SuSy, positioning it as a central regulator in source–sink dynamics and a key player in conferring abiotic stress tolerance in plants. The equilibrium between sucrose and glucose maintained by SuSy is essential for plant adaptation to heat stress. SuSy’s diverse localization within the cell and its regulation of sucrose metabolism make it indispensable to the plant’s adaptive response. Apart from its central role in metabolism, this enzyme also participates in a wide range of physiological processes, including stomatal regulation, pollen–pistil interaction, and adaptation of growth and development under environmental stress. While many other molecular players are important in these stress responses, SuSy’s distinct ability to function across multiple cellular contexts and developmental stages makes it an especially compelling focus of study. However, much of our current knowledge remains fragmented, with the molecular details of SuSy’s interactions and regulatory networks still only partially understood. Bridging these knowledge gaps will require integrated research to unravel the comprehensive mechanisms by which SuSy, in concert with other metabolic and signaling pathways, supports plant resilience under stress.

## 6 Future prospects

Future research on SuSy should focus on elucidating isoform-specific functions, regulatory mechanisms, and interactions within broader metabolic networks to enhance crops. A single gene can have different haplotypes, influencing agronomic traits in different ways. The versatility of SuSy isoforms and distinct gene types in conferring abiotic stress tolerance makes them promising candidates for crop improvement programs. Interactions between these isoforms and environmental factors create a complex metabolic regulatory network, which likely impacts plant growth and development under stress conditions. Moreover, the differential transcriptional responses of SuSy isoenzymes to environmental and metabolic stimuli are likely driven by variations in their promoter regions, an insight that opens new avenues for targeted biotechnological interventions. Notably, SuSy may play an unexpectedly important role in human nutrition: the amino acid profile of this protein, along with its high abundance in mature grains, positions it as a major contributor to lysine—a nutritionally limiting amino acid in maize kernels (Azama et al., 2003). Together with two other cytoskeletal proteins, UDPG starch glucosyl transferase and fructose 1,6-bisphosphate aldolase, SuSy supplies roughly 75% of the total lysine content in maize kernels (Koch, 2004). Thus, research in the emerging non-canonical roles of SuSy could support nutritional enhancement and stress adaptation. Field-level validation under diverse environmental conditions remains essential to translate laboratory findings into climate-resilient, high-performing cultivars. Ultimately, expanding knowledge of SuSy’s molecular mechanisms and harnessing its versatility may unlock new strategies for crop improvement, enabling plants to thrive in changing climates.
